# Serum Proteomics Distinguish Subtypes of NMO Spectrum Disorder and MOG Antibody-Associated Disease and Highlight Effects of B-Cell Depletion

**DOI:** 10.1212/NXI.0000000000200268

**Published:** 2024-06-17

**Authors:** Saurabh Gawde, Nadja Siebert, Klemens Ruprecht, Gaurav Kumar, Rose M. Ko, Kaylea Massey, Joel M. Guthridge, Yang Mao-Draayer, Patrick Schindler, Maria Hastermann, Gabriel Pardo, Friedemann Paul, Robert C. Axtell

**Affiliations:** From the Arthritis and Clinical Immunology Research Program (S.G., G.K., R.M.K., K.M., J.M.G., Y.M.-D., G.P., R.C.A.), Oklahoma Medical Research Foundation; Department of Microbiology and Immunology (S.G., R.C.A.), Oklahoma University Health Science Center; NeuroCure Clinical Research Center and Experimental and Clinical Research Center (N.S., K.R., P.S., M.H., F.P.), Max Delbrueck Center for Molecular Medicine and Charité Universitätsmedizin Berlin; and Department of Neurology (N.S., K.R., P.S., M.H., F.P.), Charité Universitätsmedizin Berlin, corporate member of Freie Universität Berlin and Humboldt-Universität zu Berlin, Germany.

## Abstract

**Background and Objectives:**

AQP4 antibody-positive NMOSD (AQP4-NMOSD), MOG antibody-associated disease (MOGAD), and seronegative NMOSD (SN-NMOSD) are neuroautoimmune conditions that have overlapping clinical manifestations. Yet, important differences exist in these diseases, particularly in B-cell depletion (BCD) efficacy. Yet, the biology driving these differences remains unclear. Our study aims to clarify biological pathways distinguishing these diseases beyond autoantibodies and investigate variable BCD effects through proteomic comparisons.

**Methods:**

In a retrospective study, 1,463 serum proteins were measured in 53 AQP4-NMOSD, 25 MOGAD, 18 SN-NMOSD, and 49 healthy individuals. To identify disease subtype-associated signatures, we examined serum proteins in patients without anti-CD20 B-cell depletion (NoBCD). We then assessed the effect of BCD treatment within each subtype by comparing proteins between BCD-treated and NoBCD-treated patients.

**Results:**

In NoBCD-treated patients, serum profiles distinguished the 3 diseases. AQP4-NMOSD showed elevated type I interferon-induced chemokines (CXCL9 and CXCL10) and TFH chemokine (CXCL13). MOGAD exhibited increased cytotoxic T-cell proteases (granzyme B and granzyme H), while SN-NMOSD displayed elevated Wnt inhibitory factor 1, a marker for nerve injury. Across all subtypes, BCD-treated patients showed reduction of B-cell–associated proteins. In AQP4-NMOSD, BCD led to a decrease in several inflammatory pathways, including IL-17 signaling, cytokine storm, and macrophage activation. By contrast, BCD elevated these pathways in patients with MOGAD. BCD had no effect on these pathways in SN-NMOSD.

**Discussion:**

Proteomic profiles show unique biological pathways that distinguish AQP4-NMOSD, MOGAD, or SN-NMOSD. Furthermore, BCD uniquely affects inflammatory pathways in each disease type, providing an explanation for the disparate therapeutic response in AQP4-NMOSD and MOGAD.

## Introduction

Neuromyelitis optica spectrum disorder (NMOSD) and myelin oligodendrocyte glycoprotein antibody-associated disease (MOGAD) are rare, yet severe, autoimmune diseases of the CNS, which predominantly affect the spinal cord and optic nerves.^1,2^ Both diseases typically exhibit a relapsing pattern, marked by recurrent optic neuritis (ON) and transverse myelitis (TM) attacks, which can lead to visual impairment and motor disability. Despite the similarities in presentation, important clinical distinctions exist between these diseases, highlighting the importance of accurate diagnosis for effective care of patients.

For NMOSD, diagnosis relies on clinical presentation, including ON and longitudinally extensive TM lesions spanning over 3 vertebral segments of the spinal cord.^1^ The majority of patients with this clinical phenotype exhibit autoreactive immunoglobulin (Ig)G antibodies against aquaporin-4 (AQP4-NMOSD), and seropositivity for AQP4-IgG is an additional diagnostic criterion for NMOSD.^1,3-5^ However, some patients meeting the clinical NMOSD criteria do not have AQP4-IgG. Although some of these patients were found to have IgG antibodies against MOG (MOG-IgG), it soon turned out that detection of MOG-IgG defines a distinct disease entity of its own right, now recognized as MOGAD, whose clinical features partially overlap with that of AQP4-NMOSD.^2^

There is a small subset of patients meeting the NMOSD clinical criteria^1^ that are seronegative for AQP4-IgG and MOG-IgG, which are termed seronegative (SN)-NMOSD. Distinct clinical and demographic differences have been established between AQP4-NMOSD and MOGAD.^6-9^ However, comparative studies with SN-NMOSD are currently limited.^10^

An important contrast between AQP4-NMOSD and MOGAD lies in the effectiveness of B-cell depleting therapies (BCD). Multiple studies have demonstrated that BCD with anti-CD20 monoclonal antibodies^11-14^ almost completely eliminates relapses in AQP4-NMOSD patients and BCD with anti-CD19 antibodies has gained regulatory approval for NMOSD.^15,16^ However, in patients with MOGAD, the efficacy of anti-CD20 is lower when compared with AQP4-NMOSD, and some patients may experience relapses despite the total depletion of B cells.^17,18^ Limited information is available regarding BCD in SN-NMOSD; however, anti-CD20 may reduce relapse rates in these patients.^13,19^

Our previous research has illustrated the utility of serum proteins in uncovering underlying pathologic mechanisms in patients with various neurologic autoimmune diseases and in discerning varied responses to treatments.^20-22^ Hence, the primary objective of this study was to pinpoint disease-related serum proteins and gain insights into the effect of B-cell depleting treatment on patients with AQP4-NMOSD, MOGAD, and SN-NMOSD.

## Methods

### Patient Cohort

Serum samples were obtained from 96 patients and 49 healthy controls at the Oklahoma Medical Research Foundation Multiple Sclerosis Center of Excellence and Charité Universitätsmedizin Berlin between 2013 and 2020. All patients were diagnosed using the Wingerchuk criteria for NMOSD diagnosis.^1^ Relapses were further determined using both clinical assessment and presence of new gadolinium-enhanced lesions using MRI.^1^ Healthy controls were defined as not being diagnosed with inflammatory neurologic or autoimmune diseases (including meningitis, MS, NMOSD, MOGAD, rheumatoid arthritis, systemic lupus erythematosus, and myasthenia gravis). Sera from all patients were tested for AQP4-IgG and MOG-IgG using cell-based assays.^23,24^ Of these patients, 53 were AQP4-IgG–positive, 25 were MOG-IgG–positive, and 18 were seronegative for both AQP4-IgG and MOG-IgG. Patients exhibiting low serum titers (below 1:100) of MOG-IgG, but presenting with additional supportive clinical features, were diagnosed with MOGAD, as outlined previously.^2^ All SN-NMOSD met the International Panel for Neuromyelitis Optica (NMO) Diagnosis criteria.^1^ The patient population had varying histories of DMT use before blood draw. Demographic information for these patients and healthy controls is summarized in Table 1. The races of the participants were self-identified.

**Table 1:** Demographics of Patients and Healthy Controls

	Disease type			Healthy	
	AQP4-NMOSD	MOGAD	SN-NMOSD	Controls	*p* Value
Number	53	25	18	49	NA
Age (mean ± SD)	50.19 ± 14.71	43.12 ± 15.14	48.72 ± 12.94	39.08 ± 14.07	0.0012^a^
Sex: female:male	49:4	15:10	12:6	36:13	0.0052^b^
Race: number (%)					0.3593^b^
White	36 (67.9%)	23 (92.0%)	15 (83.3%)	31 (63.3%)	
Black	6 (11.3%)	0 (0%)	1 (5.6%)	6 (12.2%)	
Asian	1 (1.9%)	0 (0%)	0 (0%)	4 (8.1%)	
Native American	1 (1.9%)	0 (0%)	1 (5.6%)	6 (12.2%)	
Unknown	9 (16.9%)	2 (8.0%)	1 (5.6%)	2 (4.1)%	
DMTs: number (%)					NA
BCD	24 (45.3%)	8 (32.0%)	5 (27.8%)	NA	
Non-BCD	29 (54.7%)	17 (68.0%)	13 (72.2%)	NA	
Disease activity: number (%)					NA
Active	2 (3.8%)	3 (12.0%)	2 (11.1%)	NA	
Stable	51 (96.2%)	22 (88.0%)	16 (88.9%)	NA	

All BCD patients were treated with rituximab.

All non-BCD patients were treated with no treatment, azathioprine, belimumab, glatiramer acetate, mycophenolate mofetil, methotrexate, plasmapheresis, IV immunoglobulin, and tocilizumab.

Active includes patients with enhancing lesions up to 3 mo before blood draw.

Stable patients include patients without enhancing lesions up to 3 mo before blood draw.

a *p*-value determined by one-way ANOVA.

b *p*-value determined by χ^2^.

### Standard Protocol Approvals, Registrations, and Patient Consents

Informed consent was obtained from individuals before participation in the study, which was approved by the Oklahoma Medical Research Foundation and Charité Universitätsmedizin Berlin's institutional review boards.

### Protein Quantification

Proteins were measured in serum samples using Olink Explore 1,536 from Olink Proteomics which combines the proximity extension assay (PEA) technology with next-generation sequencing technology,^25^ blinded from the clinical information linked to the samples. The complete library contains antibodies targeting 1,472 proteins, of which 1,463 are unique proteins. There are 2 antibodies targeting each unique protein, which are labelled with 2 separate, unique oligonucleotide PEA probes, which have complementary sequences. The conjugated antibodies are then mixed into 4 separate 384-plex panels, which contain 372 proteins and 12 internal controls each, which are used for quality control and normalization. These 4 panels are focused on inflammation, oncology, cardiometabolic, and neurology proteins, respectively. Patient samples were randomized and incubated overnight with antibodies conjugated to oligonucleotide PEA probes at 4°C. After antibody binding and oligonucleotide annealing, an extension and preamplification mix were added to the samples at room temperature for PCR amplification. PCR amplicons obtained were then pooled and subjected to another PCR amplification step after addition of individual sample index sequences. After another pooling of samples, bead purification and QC of the generated libraries were performed. Sequencing was then performed using Illumina's NovaSeq 6000 instrument. A quality control (QC) and normalization process was then performed to translate barcode sequence counts into normalized protein expression units. In total, 1,461 proteins successfully passed quality control and were subsequently used for the analysis.

### Bioinformatics and Pathway Analysis

Proteins were mapped to biological pathways using Ingenuity Pathway Analysis (IPA) (QIAGEN Inc.) and the STRING protein database (Table 2).^26^ B-cell–associated proteins were identified by mapping to the Human Protein Atlas (HPA) database.^27^

**Table 2:** Pathway Analysis for Protein Clusters Using STRING Protein Database

Cluster	Pathway database	Pathway	Proteins
Cluster 1	KEGG pathways	Apoptosis	GZMB, CTSC, TP53, CASP10, CASP8, PARP1, DFFA
Cluster 2	KEGG pathways	MAPK signaling	IL1B, FGF2, EGF, RASA1, TGFA, PDGFB, CRKL, MAP3K5, IRAK1, STK4, MAP2K6
	WikiPathways	IL-18 signaling	CA11, MMP8, IL1B, IL18RAP, CXCL8, BID, IRAK1, MMP9, RANGAP1, CCL3
Cluster 3	KEGG pathways	TNF signaling	CX3CL1, IL15, CCL20, FIGF, FAS
	Reactome	L1CAM interactions	NCAN, CHL1, NFASC, NRP2, ITGB1, CNTN1, NCAM1
Cluster 4	KEGG pathways	Viral protein interaction with cytokine and cytokine receptor	TNFRSF1A, IL2RB, CCL22, CCL2, CCL21, TNFRSF10B, CXCL13, CXCL10, CXCL11, CCL19, CXCL9, IL20, TNFRSF1B

Abbreviations: KEGG = kyoto encyclopedia of genes and genomes; L1CAM = neural cell adhesion molecule L1; MAPK = mitogen-activated protein kinase; TNF = tumor necrosis factor.

### Statistical Analysis

Multiple *t* tests were performed using the Bonferroni and Hochberg method and corrected for covariates using the limma package. Corrected *p* values ≤0.05 were considered significant. Unsupervised hierarchical clustering was performed on serum proteins using k-means clustering, and heatmaps were generated using the ComplexHeatmap package.^28^ Data for grouped comparisons are presented as mean ± SEM, and statistical significance was determined using two-tailed Student t-tests or one-way ANOVA with Tukey or Benjamini, Krieger, and Yekutieli correction when 2 or more groups were compared. GraphPad Prism v9.5.0 and R v4.2.3 were used for statistical analyses. Statistical tests used are indicated in the methods and in the figure and table legends.

### Data Availability

All proteomic data and deidentified clinical information will be made available on request.

## Results

### Serum Proteins Distinguish AQP4-NMOSD, MOGAD, and SN-NMOSD From Healthy Individuals

**Figure 1:**
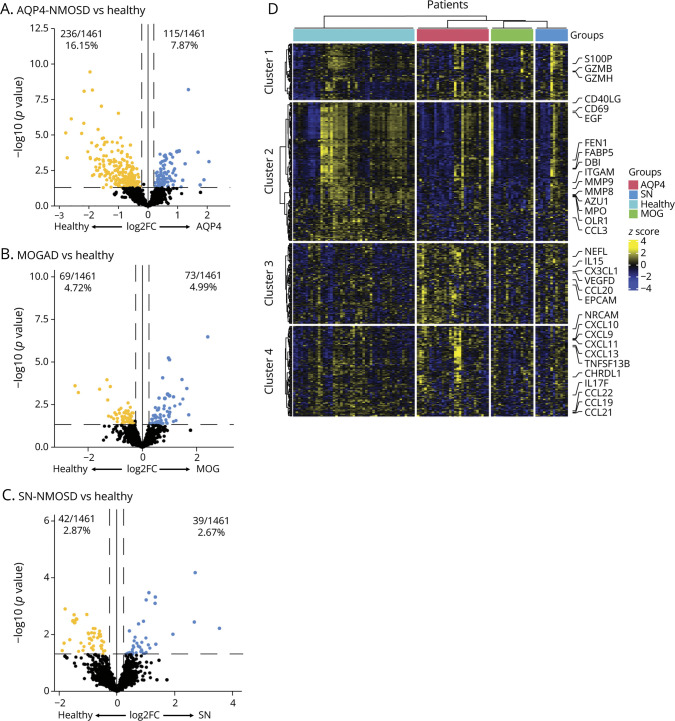
Serum Protein Profiles Distinguish NMOSD Subtypes and MOGAD That Are Not Undergoing B-Cell Depletion From Healthy Controls Comparison of serum protein profiles between (A) noBCD AQP4-NMOSD and healthy controls, (B) noBCD MOGAD and healthy controls, and (C) noBCD SN-NMOSD and healthy controls. Significant proteins were determined using unpaired *t* tests with multiple comparisons setting a *p*-value of 0.05. (D) Heatmap of hierarchical clustering of serum proteins significantly different between diseases and healthy controls. BCD = B-cell depletion; MOGAD = myelin oligodendrocyte glycoprotein antibody-associated disease; NMOSD = neuromyelitis optica spectrum disorder.

Our first goal was to identify serum proteins that distinguish AQP4-NMOSD, MOGAD, and SN-NMOSD patients from healthy donors (Table 1). In our cohort, a significant proportion of patients were on BCD, which can dramatically alter serum protein levels in patients and obscure important differences between these disease entities. Therefore, we compared serum protein profiles of the patients who were not on BCD (NoBCD) treatment with healthy controls using multiple unpaired t-tests adjusted for age, sex, race, and treatments as covariates. In noBCD AQP4-NMOSD (n = 29), 115 proteins (*p*-value <0.05) were increased and 236 proteins (*p*-value <0.05) were reduced compared with healthy controls (Figure 1A, eTable 1). In noBCD MOGAD (n = 17), 73 proteins (*p*-value <0.05) were increased and 69 proteins (*p*-value <0.05) were reduced compared with healthy controls (Figure 1B, eTable 2). In noBCD SN-NMOSD (n = 13), 39 proteins (*p*-value <0.05) were increased and 42 proteins (*p*-value <0.05) were reduced compared with healthy controls (Figure 1C, eTable 3).

Next, we performed unsupervised hierarchical clustering using k-means clustering on the significantly different serum proteins, identified above, to distinguish noBCD AQP4-NMOSD, MOGAD, and SN-NMOSD patients from healthy controls (Figure 1D). This method grouped proteins into clusters that are similar to each other in expression patterns in our cohort. The significantly different serum proteins separated into 4 clusters. Proteins from each cluster were then mapped to signaling pathways using the STRING database to identify pathways that may be relevant to each disease entity (Table 2). Proteins from cluster 1 mapped to the apoptosis pathway, including granzyme B (GZMB) and granzyme H (GZMH), which were elevated in noBCD MOGAD compared with noBCD AQP4-NMOSD and in NoBCD MOGAD and noBCD SN-NMOSD compared with healthy controls (eFigure 1A). GZMB and GZMH are produced by cytotoxic T cells and could be potential biomarkers for MOGAD.^29^

Of the proteins belonging to cluster 3, fractalkine (CX3CL1) and vascular endothelial growth factor D (VEGFD) were elevated in noBCD AQP4-NMOSD compared with noBCD MOGAD and in noBCD AQP4-NMOSD compared with healthy controls (eFigure 1B). A previous study has implicated CX3CL1, which is a chemokine for microglia, as a biomarker of AQP4-NMOSD disease activity.^30^ There is a potential role for VEGF in promoting the breakdown of the blood-brain barrier in AQP4-NMOSD^31^ and neutralizing antibodies against VEGF are being tested as a therapy for this disease.^32^

Proteins belonging to cluster 4 mapped to the viral protein interaction with cytokine and cytokine receptor pathway and contained type I interferon (IFN)-inducible chemokines CXCL9, CXCL10, and CXCL11. Levels of CXCL9, CXCL10, and CXCL11 were significantly elevated in noBCD AQP4-NMOSD compared with noBCD MOGAD and healthy controls. CXCL9 and CXCL10 levels were also elevated in noBCD AQP4-NMOSD patients compared with noBCD SN-NMOSD (eFigure 1C). Our previous studies have shown that CXCL9, CXCL10, and CXCL11 levels are increased in NMOSD patients with a high type I IFN transcriptional signature.^21^ We also observed a significant increase in the B-cell/T-follicular helper (TFH) chemokine CXCL13 in noBCD AQP4-NMOSD compared with noBCD MOGAD, noBCD SN-NMOSD, and healthy controls (eFigure 1C). Elevated levels of CXCL13 and CXCL10 have been detected in both serum and CSF of patients with NMOSD.^33^ These comparisons with healthy volunteers demonstrate that distinct biological pathways are dysregulated in each disease subtype.

### Type I IFN Chemokines Distinguish AQP4-NMOSD From MOGAD and SN-NMOSD

**Figure 2:**
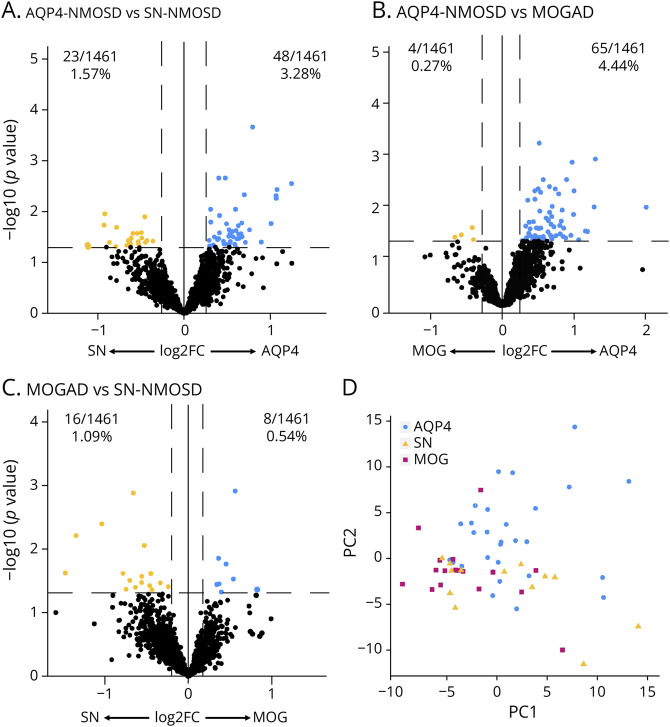
Serum Protein Profiles Distinguish AQP4-NMOSD, MOGAD, and SN-NMOSD Patients Who Are Not Undergoing B-Cell Depletion Comparison of serum protein profiles between (A) noBCD AQP4-NMOSD and noBCD SN-NMOSD, (B) noBCD AQP4-NMOSD and noBCD MOGAD, and (C) noBCD MOGAD and noBCD SN-NMOSD. Significant proteins were determined using unpaired *t* tests with multiple comparisons setting a *p*-value of 0.05. (D) Principal component analysis (PCA) of significantly different proteins between diseases. BCD = B-cell depletion; MOGAD = myelin oligodendrocyte glycoprotein antibody-associated disease; NMOSD = neuromyelitis optica spectrum disorder.

We directly compared serum protein profiles of AQP4-NMOSD, MOGAD, and SN-NMOSD patients who were not on BCD. When comparing noBCD AQP4-NMOSD with noBCD SN-NMOSD, we discovered that 48 proteins were elevated and 23 proteins were reduced (*p*-value <0.05) in AQP4-NMOSD (Figure 2A, eTable 4). The noBCD AQP4-NMOSD with noBCD MOGAD comparison identified 65 proteins that were increased and only 4 proteins were reduced (*p*-value <0.05) in AQP4-NMOSD compared with MOGAD (Figure 2B, eTable 5). Finally, we determined that 8 proteins were increased, and 16 proteins were reduced (*p*-value <0.05) in MOGAD compared with SN-NMOSD (Figure 2C, eTable 6).

Of these intercomparisons, there were 51 proteins distinct to the AQP4-NMOSD⇔MOGAD comparison, 48 proteins distinct to the AQP4-NMOSD⇔SN-NMOSD comparison, and 14 proteins distinct to the SN-NMOSD⇔MOGAD comparison (eFigure 2). Principal component analysis using these distinct proteins separated the majority of the AQP4-NMOSD patients from the other 2 disease subtypes (Figure 2D).

Features that distinguished noBCD AQP4-NMOSD from the other disease subtypes were elevated levels of the type I IFN chemokines CXCL9 and CXCL10 (eFigure 3, A and B). CXCL11 and IL-15 were increased in AQP4-NMOSD compared with MOGAD only (eFigure 3, C and D). Plexin-B2 (PLXNB2) and serine proteinase inhibitor 9 (SERPINA9) were elevated in both AQP4-NMOSD and MOGAD patients compared with SN-NMOSD (eFigure 3, E and F). Of interest, PLXNB2 and SERPINA9 are both expressed by germinal center B cells, and their upregulation could indicate involvement of germinal center B cells in the autoantibody-positive disease pathology of AQP4-NMOSD and MOGAD compared with DN NMOSD.^34-36^ Wnt inhibitory factor 1 (WIF1) levels were significantly increased in SN-NMOSD compared with AQP4-NMOSD and MOGAD patients (eFigure 3G). Dynactin-6 (DCTN6) levels were increased in SN-NMOSD compared with AQP4-NMOSD only (eFigure 3H). WIF1 and DCTN6 are upregulated in response to spinal cord injury and associated with regenerative response in mice.^37^ Therefore, these proteins could indicate recovery from spinal injury in patients with NMOSD. Collectively, these findings illustrate that serum proteins, particularly type I IFN chemokines, distinguish AQP4-NMOSD from MOGAD and SN-NMOSD.

### Differential Effects of B-Cell Depletion on AQP4-NMOSD, MOGAD, and SN-NMOSD

B-cell depletion with anti-CD20 antibody is a widely used treatment for NMOSD.^38^ Nevertheless, it is evident that the AQP4-NMOSD and MOGAD exhibit distinct responses to this therapy. Although reports have highlighted the high efficacy of BCD in AQP4-NMOSD patients, a notable proportion of patients with MOGAD continue to experience relapses despite this treatment.^17,18,39^ The effectiveness of BCD in SN-NMOSD remains uncertain.^19^ Our cohort included a substantial number of patients from each disease entity undergoing BCD treatment (Table 1). Consequently, we conducted a comparative analysis to explore similarities and differences in serum profiles between patients who received BCD and those who did not within each disease subtype.

**Figure 3:**
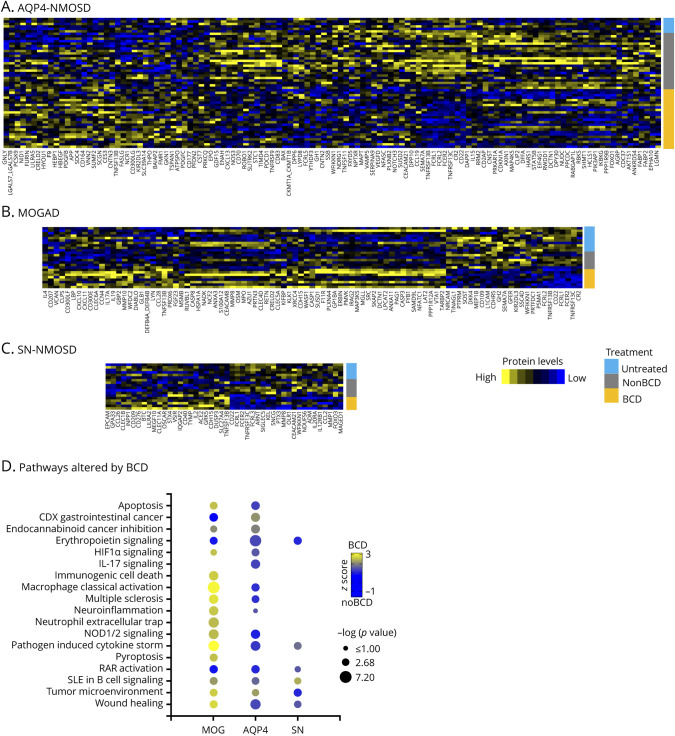
Effect of B-Cell Depletion on Serum Proteomic Signatures in AQP4-NMOSD, MOGAD, and SN-NMOSD Patients Heatmap depicts supervised clustering of the proteins significantly altered in BCD vs NoBCD in (A) AQP4-NMOSD, (B) MOGAD, and (C) SN-NMOSD. The significant proteins used to generate the heatmaps were determined using unpaired t-tests with multiple comparisons setting a *p*-value of 0.05. (D) Ingenuity Pathway Analysis (IPA) of pathways altered by BCD vs NoBCD in each disease. Z-scores and -log10*p* values were determined using IPA. BCD = B-cell depletion; MOGAD = myelin oligodendrocyte glycoprotein antibody-associated disease; NMOSD = neuromyelitis optica spectrum disorder.

In AQP4-NMOSD, there were 37 proteins with increased levels and 81 proteins with decreased levels (*p*-value <0.05) in BCD-treated patients (n = 24) when compared with those who did not receive BCD (n = 29) (eTable 7). In MOGAD, we observed 71 proteins with elevated levels and 24 proteins with reduced levels (*p*-value <0.05) in BCD-treated patients (n = 8) in contrast to noBCD patients (n = 17) (eTable 8). For SN-NMOSD, there were 24 proteins with increased levels and 22 proteins with decreased levels (*p*-value <0.05) in BCD-treated patients (n = 5) compared with those not receiving BCD (n = 13) (eTable 9). Moreover, we generated heatmaps using these proteins, revealing distinctive serum protein profiles for BCD-treated patients within each of the disease subgroups (Figure 3, A–C).

We assessed the similarities and differences between the BCD vs NoBCD comparisons for the disease types, which are depicted in a Venn diagram (eFigure 4A). These comparisons revealed that BCD induced distinct proteomic changes in AQP4-NMOSD, MOGAD, and SN-NMOSD diseases. In fact, we identified only 6 common proteins of a total of 237 that were altered by BCD in all 3 NMOSD subtypes (eFigure 4B). Among these common proteins, 5 exhibited reduced levels in BCD-treated patients: FCER2, TNFRSF13C, WFIKNN1, FCRL1, and CD22. The HPA, a database comprising publicly available gene-expression and proteomic data, indicated that 4 (CD22, TNFRSF13C, FCRL1, and FCER1) of these 5 proteins are preferentially expressed by B cells, suggesting effective B-cell depletion in these patients. In addition, TNFSF13B, also known as BAFF, was significantly elevated in all NMOSD subtypes. The elevation of BAFF has been observed in BCD treatment for various diseases, including NMOSD.^30^

To gain a more comprehensive understanding of the influence of BCD on NMOSD, we used IPA to pinpoint the biological pathways affected by BCD. This analysis underscored the distinct effects of BCD on each disease entity. Notably, macrophage classical activation, neutrophil extracellular trap formation, and cytokine storm signaling were all upregulated in patients with MOGAD because of BCD, whereas in AQP4-NMOSD patients, these pathways were downregulated (Figure 3D).

**Figure 4:**
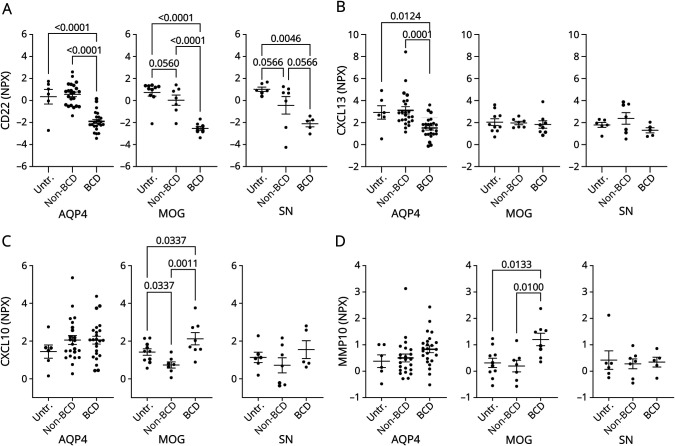
Effect of B-Cell Depletion on Individual Serum Proteins Comparison of serum levels of (A) CD22, (B) CXCL13, (C) CXCL10, and (D) MMP10 between untreated (UnTx), non-BCD (nonBCD), and BCD-treated patients for each disease type. One-way ANOVA test with a Benjamini, Krieger, and Yekutieli correction for multiple comparisons was used.

We next compared the levels of key serum proteins in patients who were BCD-treated, non-BCD–treated, or untreated at the time of blood draw. Specifically, we compared serum levels of CD22 (a B-cell marker), CXCL13, and CXCL10 (markers of cytokine storm) and MMP10 (a metalloprotease expressed by activated macrophages). The results showed that CD22 levels were significantly reduced by BCD in all diseases (Figure 4A). Moreover, CD22 levels did not exhibit significant differences among the BCD-treated disease types, suggesting an equivalent reduction in B cells across these conditions (eFigure 5). Notably, we observed that CXCL13 levels were decreased by BCD in AQP4-NMOSD patients compared with both untreated and non-BCD–treated patients (Figure 4B). However, BCD did not lead to changes in CXCL13 levels in MOGAD or SN-NMOSD patients. Conversely, CXCL10 levels were significantly elevated in patients with MOGAD who underwent BCD compared with untreated and non-BCD–treated patients (Figure 4C). Of interest, non-BCD–treated MOGAD patients exhibited lower serum CXCL10 levels compared with untreated patients. Levels of CXCL10 were unaffected by BCD in AQP4-NMOSD and SN-NMO patients. Similarly, MMP10 was significantly elevated in the BCD-treated MOGAD patients compared with untreated and non-BCD–treated patients (Figure 4D). MMP10 was not altered by BCD in AQP4-NMOSD or SN-NMOSD. These findings underscore the distinct biological effects of BCD on each disease type.

## Discussion

Our study's initial objective was to compare the serum proteomes of AQP4-NMOSD, MOGAD, and SN-NMOSD with those of healthy individuals to provide insights into the underlying pathologic processes in these diseases. Given the significant effect of B-cell depletion on serum proteins, we specifically compared patients who were not receiving BCD to identify disease-specific signatures. These investigations revealed that each disease subtype displayed a unique protein signature, distinguishing them from the other subtypes and healthy individuals. Notably, the AQP4-NMOSD patients displayed a more unique proteomic signature, while the signatures of MOGAD and SN-NMOSD patients were relatively more similar to each other. Studies have shown that MOG antibodies fluctuate between detectable and undetectable levels during relapses and remissions,^40^ which is not as clearly the case for AQP4 antibodies.^41^ Therefore, we cannot exclude that some of the SN-NMOSD patients are MOGAD but had undetectable MOG antibodies at the time of autoantibody testing.

Specifically, AQP4-NMOSD patients exhibited elevated levels of a B-cell/T-follicular helper (TFH) chemokine, CXCL13, and the type I IFN-induced chemokines (CXCL11, CXCL10, and CXCL9) in comparison with the other disease subsets and healthy individuals. Previous reports have established links between type I IFN signatures and CXCL13 levels with clinical measures in patients with NMOSD.^21,42^ Our data now indicate that both type I IFN activity and B-cell activity may be significant pathologic mechanisms in AQP4-NMOSD, but they may not play an equally significant role in MOGAD or SN-NMOSD patients.

Patients with MOGAD exhibited elevated levels of granzymes in comparison with the other disease subtypes and healthy individuals. Granzymes are serine proteases released by cytotoxic immune cells, facilitating direct killing of target cells. The specific immune cell type responsible for releasing these granzymes in MOGAD remains unclear and could potentially be either cytotoxic CD8 cells or TH17 cells. CD8^+^ T cells expressing GZMH have been detected in postmortem lesions and in the peripheral blood of patients with MOGAD.^29,43^ However, in MOGAD, the most abundant T cells in lesions are CD4 T cells, and studies have shown that human CD4 cells polarized toward the TH17 phenotype express granzymes and are capable of directly targeting and killing oligodendrocytes, which express MOG.^44^

SN-NMOSD patients exhibited elevated levels of WIF1 and DCTN6 compared with the other disease subtypes, although not compared with healthy individuals. WIF1 acts as an inhibitor of the Wnt signaling pathway. Studies in spinal cord injury models have indicated that Wnt signaling can influence axonal regeneration after injury.^45^ Similarly, DCTN6 has been associated with spinal cord regeneration in mice.^37^ Hence, the upregulation of WIF1 and DCTN6 may significantly affect CNS regenerative mechanisms in SN-NMOSD when compared with AQP4-NMOSD and MOGAD.

The effectiveness of BCD with anti-CD20 treatment varies depending on the subtype of NMOSD. It has shown high efficacy in AQP4-NMOSD^11-14^ but has limited effectiveness in MOGAD.^17,18^ The effectiveness of BCD in SN-NMOSD is currently uncertain.^13^ Consequently, the second objective of our study was to investigate whether BCD treatment leads to distinct alterations in serum proteomes for each NMOSD subtype.

Within our patient cohort, we identified several proteins related to cytokine storm, macrophage activation, neutrophil activity, and IL-17 signaling pathways that were elevated in patients with MOGAD undergoing BCD. By contrast, these pathways were downregulated in AQP4-NMOSD patients receiving BCD. These observations point to varying effects of BCD in AQP4-NMOSD and MOGAD. Notably, the re-emergence of memory B cells after rituximab treatment corresponded to 92.5% of breakthrough relapses in AQP4-NMOSD. This stands in contrast to MOGAD, where B-cell repopulation was associated with only 20% of relapses.^18^ Overall, these findings suggest that B cells may serve distinct roles in AQP4-NMOSD and MOGAD. Indeed, they are clearly pathogenic in AQP4-NMOSD but might exhibit regulatory properties in MOGAD.

Although the function of B cells in MOGAD is not yet fully elucidated, our data from human MOGAD align with studies conducted on mice with MOG_35-55_-peptide-induced experimental autoimmune encephalomyelitis (EAE). Notably, in this particular EAE model, much like MOGAD, B-cell depletion is not effective in treating mice; instead, it can exacerbate disease activity and notably increase inflammatory pathways.^46^ The parallels between MOGAD and MOG-induced EAE regarding the effects of BCD on elevating inflammatory pathways suggest that B cells may play a regulatory role in this disease subtype and underscore the translational relevance of the MOG-EAE model.

Despite the overlap in clinical presentation between AQP4-NMOSD and MOGAD, recent research efforts have advocated for classifying patients with MOG antibodies as a distinct disease, separate from NMOSD.^2,47^ Adding to the complexity of NMOSD diagnosis and clinical care, the extent of similarity or difference between SN-NMOSD patients and those with AQP4-NMOSD or MOGAD remains unknown. Our studies have now identified proteomic differences in the AQP4-NMOSD, SN-NMOSD, and MOGAD that link to potential pathogenic functions. Furthermore, our study provides a potential explanation for why anti-CD20 BCD is not as effective in patients with MOGAD compared with AQP4-NMOSD patients. These key observations provide further evidence that the AQP4-NMOSD and MOGAD are distinct diseases and should be treated accordingly.

Our proteomic profiling also suggests that SN-NMOSD is more similar to MOGAD than to AQP4-NMOSD. Of interest, previous studies have identified that female sex and non-White races are demographic features linked to susceptibility and severity of AQP4-NMOSD.^48-50^ By contrast, the demographics of MOGAD show no sex bias and are predominantly composed of individuals of the White race compared with AQP4-NMOSD.^5,51^ In our cohort, we observed a notable predominance of women in AQP4-NMOSD compared with both MOGAD and SN-NMOSD. For race in our cohort, we found a trend for an increased representation of Black individuals among AQP4-NMOSD patients compared with both MOGAD and SN-NMOSD patients (Table 1). These demographic findings provide further evidence that SN-NMOSD may be more closely related to MOGAD than to AQP4-NMOSD.

NMOSD and MOGAD are rare diseases, making studies that compare disease subtypes inherently challenging. A limitation of our study is the absence of longitudinal serum samples to monitor the effect of BCD on patients with AQP4-NMOSD, MOGAD, and SN-NMOSD over time. Consequently, we used a cross-sectional study design to compare patients receiving BCD treatment with those who are not. Despite this constraint, our study successfully identified serum proteins linked to biological pathways that provide insights into the variations in clinical responses to BCD among AQP4-NMOSD, MOGAD, and SN-NMOSD. To corroborate the effects of BCD observed in our study, future research using longitudinal serum samples is imperative.

## Appendix Authors

**Table:**

Name	Location	Contribution
Saurabh Gawde, PhD	Arthritis and Clinical Immunology Research Program, Oklahoma Medical Research Foundation; Department of Microbiology and Immunology, Oklahoma University Health Science Center	Drafting/revision of the manuscript for content, including medical writing for content; major role in the acquisition of data; study concept or design; analysis or interpretation of data
Nadja Siebert, MD	NeuroCure Clinical Research Center and Experimental and Clinical Research Center, Max Delbrueck Center for Molecular Medicine and Charité Universitätsmedizin Berlin; Department of Neurology, Charité Universitätsmedizin Berlin, corporate member of Freie Universität Berlin and Humboldt-Universität zu Berlin, Germany	Major role in the acquisition of data
Klemens Ruprecht, MD	NeuroCure Clinical Research Center and Experimental and Clinical Research Center, Max Delbrueck Center for Molecular Medicine and Charité Universitätsmedizin Berlin; Department of Neurology, Charité Universitätsmedizin Berlin, corporate member of Freie Universität Berlin and Humboldt-Universität zu Berlin, Germany	Drafting/revision of the manuscript for content, including medical writing for content; major role in the acquisition of data; analysis or interpretation of data
Gaurav Kumar, PhD	Arthritis and Clinical Immunology Research Program, Oklahoma Medical Research Foundation	Major role in the acquisition of data
Rose M. Ko, PhD	Arthritis and Clinical Immunology Research Program, Oklahoma Medical Research Foundation	Drafting/revision of the manuscript for content, including medical writing for content; major role in the acquisition of data
Kaylea Massey, BSc	Arthritis and Clinical Immunology Research Program, Oklahoma Medical Research Foundation	Major role in the acquisition of data
Joel M. Guthridge, PhD	Arthritis and Clinical Immunology Research Program, Oklahoma Medical Research Foundation	Major role in the acquisition of data
Yang Mao-Draayer, MD, PhD	Arthritis and Clinical Immunology Research Program, Oklahoma Medical Research Foundation	Drafting/revision of the manuscript for content, including medical writing for content; analysis or interpretation of data
Patrick Schindler, MD	NeuroCure Clinical Research Center and Experimental and Clinical Research Center, Max Delbrueck Center for Molecular Medicine and Charité Universitätsmedizin Berlin; Department of Neurology, Charité Universitätsmedizin Berlin, corporate member of Freie Universität Berlin and Humboldt-Universität zu Berlin, Germany	Drafting/revision of the manuscript for content, including medical writing for content; major role in the acquisition of data; analysis or interpretation of data
Maria Hastermann, MD, PhD	NeuroCure Clinical Research Center and Experimental and Clinical Research Center, Max Delbrueck Center for Molecular Medicine and Charité Universitätsmedizin Berlin; Department of Neurology, Charité Universitätsmedizin Berlin, corporate member of Freie Universität Berlin and Humboldt-Universität zu Berlin, Germany	Major role in the acquisition of data
Gabriel Pardo, MD	Arthritis and Clinical Immunology Research Program, Oklahoma Medical Research Foundation	Drafting/revision of the manuscript for content, including medical writing for content; major role in the acquisition of data
Friedemann Paul, MD	NeuroCure Clinical Research Center and Experimental and Clinical Research Center, Max Delbrueck Center for Molecular Medicine and Charité Universitätsmedizin Berlin; Department of Neurology, Charité Universitätsmedizin Berlin, corporate member of Freie Universität Berlin and Humboldt-Universität zu Berlin, Germany	Drafting/revision of the manuscript for content, including medical writing for content; major role in the acquisition of data; study concept or design; analysis or interpretation of data
Robert C. Axtell, PhD	Arthritis and Clinical Immunology Research Program, Oklahoma Medical Research Foundation; Department of Microbiology and Immunology, Oklahoma University Health Science Center	Drafting/revision of the manuscript for content, including medical writing for content; major role in the acquisition of data; study concept or design; analysis or interpretation of data

## Glossary

AQP4-NMOSD: AQP4 antibody-positive NMOSD
BCD: B-cell depletion
EAE: experimental autoimmune encephalomyelitis
HPA: Human Protein Atlas
IPA: Ingenuity Pathway Analysis
MOGAD: myelin oligodendrocyte glycoprotein antibody-associated disease
NMOSD: neuromyelitis optica spectrum disorder
ON: optic neuritis
PEA: proximity extension assay
TM: transverse myelitis
